# Heterogeneity of treatment responses in rheumatoid arthritis using group based trajectory models: secondary analysis of clinical trial data

**DOI:** 10.1186/s41927-023-00348-5

**Published:** 2023-09-25

**Authors:** Fowzia Ibrahim, Ian C Scott, David L Scott, Salma Ahmed Ayis

**Affiliations:** 1 Centre for Rheumatic Diseases, Department of Inflammation Biology, School of Immunology and Microbial Sciences, Faculty of Life Sciences and Medicine, King’s College London Cutcombe Road, London, SE5 9RJ UK; 2 Primary Care Centre Versus Arthritis, School of Medicine, Keele University, Keele, UK; 3 Haywood Academic Rheumatology Centre, Midlands Partnership University NHS Foundation Trust, High Lane, Burslem, Staffordshire, UK; 4 School of Life Course and Population Sciences, King’s College London, London, UK

**Keywords:** Arthritis, rheumatoid, Clinical trial, Treatment, Statistics

## Abstract

**Background:**

Traditionally rheumatoid arthritis (RA) trials classify patients as responders and non-responders; they ignore the potential range of treatment responses. Group Based Trajectory Models (GBTMs) provide a more refined approach. They identify patient subgroups with similar outcome trajectories. We used GBTMs to classify patients into subgroups of varying responses and explore factors associated with different responses to intensive treatment in a secondary analysis of intensive treatment in the TITRATE clinical trial.

**Methods:**

The TITRATE trial enrolled 335 patients with RA: 168 patients were randomised to receive intensive management, which comprised monthly assessments including measures of the disease activity score for 28 joints (DAS28), treatment escalation when patients were not responding sufficiently and psychosocial support; 163 of these patients completed the trial. We applied GBTMs to monthly DAS28 scores over one year to these patients who had received intensive management. The control group had standard care and were assessed every 6 months; they had too few DAS28 scores for applying GBTMs.

**Results:**

GBTMs identified three distinct trajectories in the patients receiving intensive management: good (n = 40), moderate (n = 76) and poor (n = 47) responders. Baseline body mass index (BMI), disability, fatigue and depression levels were significantly different between trajectory groups. Few (10%) good responders were obese, compared to 38% of moderate, and 43% of poor responders (*P* = 0.002). Few (8%) good responders had depression, compared to 14% moderate responders, and 38% poor responders (*P* < 0.001). The key difference in treatments was using high-cost biologics, used in only 5% of good responders but 30% of moderate and 51% of poor responders (*P* < 0.001). Most good responders had endpoint remissions and low disability, pain, and fatigue scores; few poor responders achieved any favourable outcomes.

**Conclusion:**

GBTMs identified three trajectories of disease activity progression in patients receiving intensive management for moderately active RA. Baseline variables like obesity and depression predicted different treatment responses. Few good responders needed biologic drugs; they responded to conventional DMARDs alone. GBTMs have the potential to facilitate precision medicine enabling patient-oriented treatment strategies based on key characteristics.

**Registration:**

TITRATE Trial ISRCTN 70160382.

## Introduction

Trials in rheumatoid arthritis (RA) traditionally classify patients into responders and non-responders. This approach reflects European League Against Rheumatism (EULAR) and American College of Rheumatology (ACR) response criteria [1]. It identifies effective new treatments and compares efficacies of existing treatments. However, wider categorisation of responses may be preferable for individualising care using treat-to-target [2] approaches.

Latent class models have been used to analyse long-term observational studies of RA to identify sub-groups of patients for some time. They have evaluated changes in disability [3, 4], psychological distress [5] and disease activity [6]; between three and six distinct trajectories were reported in these different studies. More recently, Bykerk et al. extended using disease trajectories to identify different patient groups in a clinical trial [7]. Their post hoc analysis of patients receiving Tofacitinib over 2 years in a phase III trial identified five different trajectories in the disease activity score for 28 joints using the erythrocyte sedimentation rate (DAS28-ESR). When latent class models were applied to patients with early RA managed by treat-to-target approaches enrolled in the DREAM and BARFOT registries [3, 8], three distinct responder groups were identified from changes in DAS28-ESR scores. The best responders achieved remissions whilst the worst responders had persistently active disease. There was a similar pattern of three responder groups in treated early RA patients assessed with the Simplified Disease Activity Index [9].

Experience in these observational studies and the analysis of existing clinical trial data suggests that when patients with RA are managed using treat-to-target approaches, identifying groups with different outcome trajectories gives useful information about the benefits of active treatment. We examined this concept in a post hoc analysis of patients randomised to receive intensive treatment in the TITRATE trial [10], which individualised treatments with the goal of achieving remission at 12 months. Our analysis addressed three issues: first, the practicality of using latent class modelling in trial patients followed over 12 months and the number of distinct trajectories identified; second, baseline factors influencing membership of the different trajectories; and third, the effects of different components of intensive management on the membership of different trajectories.

## Methods

### Patients studied

The TITRATE trial [10] enrolled 335 patients with RA from 39 UK centres; 168 were randomised to receive intensive management. The aim of the original trial was to test the hypothesis that intensive management resulted in more remissions at 12 months than standard care in patients with moderate RA. The trial confirmed this hypothesis [10].

TITRATE enrolled patients aged ≥ 18 years who met the 1987 American College of Rheumatology (ACR) or 2010 European League Against Rheumatism (EULAR)/ACR classification criteria for RA [11, 12]. They had received at least 6 months conventional disease-modifying anti-rheumatic drugs (DMARDs), were currently receiving at least one DMARD, and had moderate/intermediate disease activity (DAS28-ESR 3.2–5.1). Patients were excluded if they had co-morbidities making intensive treatment inadvisable, had failed five or more conventional DMARDs, had taken biologics, or had extensive joint damage. 168 patients were randomised to intensive management: 5 patients withdrew after screening and randomisation assessments; we therefore analysed the 163 with at least one follow-up visit.

Patients enrolled to the comparator standard care treatment arm are not included in the analysis for several reasons. First, they only had DAS28-ESR scores measured every six months and constructing latent class trajectories in six monthly data is not comparable to comparing monthly changes. Second, the main trial paper compared the two treatment groups at the trial endpoint, showing significant differences between groups; there are cogent reasons not to continually reanalyse trial data using different approaches to compare treatments. Third, our research question is whether the simple approach of classifying treatment effects into responders and non-responders is ideal, or whether using latent class trajectory modelling identifies more groups in patients receiving active treatment. Investigating this aim only requires evaluating the actively treated patients.

### Intensive treatment

Intensive management was delivered by trained rheumatology nurses or comparable healthcare professionals who had all been specifically trained to deliver the management regimen. Decisions about treatments were made by the whole clinical team looking after the patients. A wide range of considerations were involved in management decisions and the DAS28-ESR was used as part of this process. Treatment with conventional DMARDs and biologics was optimised following a treatment algorithm which included also giving intra-muscular (IM) steroid injections. The nurses also provided supportive management for pain and fatigue. HAQ, pain and fatigue were assessed 6 monthly; they changed significantly between baseline and 12 months. PHQ-9 was only measured at baseline. These assessments were not used for decisions about intensive management. Full details are provided in the trial report [10] .

### Outcome measures

DAS28-ESR and its components were measured monthly. This index involves making a calculation based on four standard clinical assessments: tender joint counts for 28 joints, swollen joint counts for 28 joints, the patient global assessment on a 100 mm visual analogue score and the ESR. Further details of the DAS28-ESR score and its variants are outlined by Van Riel and Renskers [13]. C-reactive protein (CRP), assessor global assessments pain and fatigue (on 100 mm visual analogue scales) and function measured by the Health Assessment Questionnaire (HAQ) were assessed 6 monthly. Demographic details, smoking habits, body mass index (BMI) and mood (Patient Health Questionnaire-9, (PHQ9)) were assessed at baseline.

One assessment approach that was not used in the TITRATE trial was the American College of Rheumatology responder criteria. The main reason for not evaluating these is that they are not employed in routine clinical settings in England. In addition, the trial assessed patients with moderate disease activity and the value of these criteria in such patients is uncertain.

### Statistical methods

Baseline information were summarised using the mean, with accompanying standard deviations (SD) for continuous variables, while binary or categorical variables were summarised using frequency and percentage.

Group Based trajectory Models (GBTMs) were used to identify clusters of disease activity trajectories over 12 months following the commencement of treatment [14]. Latent Class Analysis (LCA) is a statistical measurement model in which individuals can be classified into mutually exclusive and exhaustive groups or latent classes, based on their pattern of response on a variable or a set of variables. In our model longitudinal data from DAS28-ESR was used to generate the groups; it was a different approach to simply dividing patients into groups based on 12-month assessments, or any specific time point’s assessment. The primary outcome measure used to derive the trajectories was DAS28-ESR and no other covariates were included. GBTMs are likelihood-based methods, which are valid using only observed data, under a missing-at-random assumption. When this approach is taken patients do not form two clearly defined groups of responders and non-responders: instead GBTMs identified three different developmental trajectories over the 12 months of the trial; the model took account of all the repeated measurements, rather than one single time point, at any time during the follow-up period or simply using the final time point at end of the study.

The best choice of the number of latent classes (3 classes vs. 4 classes) was made using Bayesian Information Criterion (BIC); the average posterior probability of class membership exceeding 0.7, entropy that indexes classification accuracy, with values closer to 1 indexing greater precision [15]. In addition, judgment that classes are clinically meaningful and represent distinct features was taken into consideration supporting the formal statistical tests [16].

Associations of trajectory classes with outcomes, baseline covariates, RA medication prescribing, alcohol and smoking status were assessed either using analysis of variance or Fisher’s exact test as appropriate for the variable being compared. We used Spearman’s correlation to test the association between the variables before fitting the multivariable model. We used change in outcome of interest at 12-months instead of actual score at 12-months to adjust for baseline values. As expected, change in DAS28-ESR was associated with all three endpoints: the strongest correlation was with change in pain (correlation coefficient (r) = 0.49), followed by change in HAQ (r = 0.33) and change in fatigue (r = 0.28). Since DAS28-ESR was used to derive the trajectory grouping, it was not included in the multivariable model. In this model, demographic variables (age, gender, ethnicity, smoking history and disease duration), baseline body mass index (BMI), baseline PHQ-9 and change in HAQ, pain, and fatigue, respectively were included. Forward stepwise multinomial logistic regression with trajectory groups as the dependent variable was used, p-value was set to 5%, which meant that variables with a significance level p ≥ 0.05 were removed from the model. Multinomial logit model was chosen as the dependent variable is categorical with more than two levels. In addition, a sensitivity analysis was done, where the p-value was set to 15%; the results were the same.

All analyses were carried out using STATA (StataCorp. 2017. Stata Statistical Software: Release 16. College Station, TX: StataCorp LLC). *P*-values < 0.05 were considered statistically significant.

## Results

### Trajectory groups

**Fig. 1:**
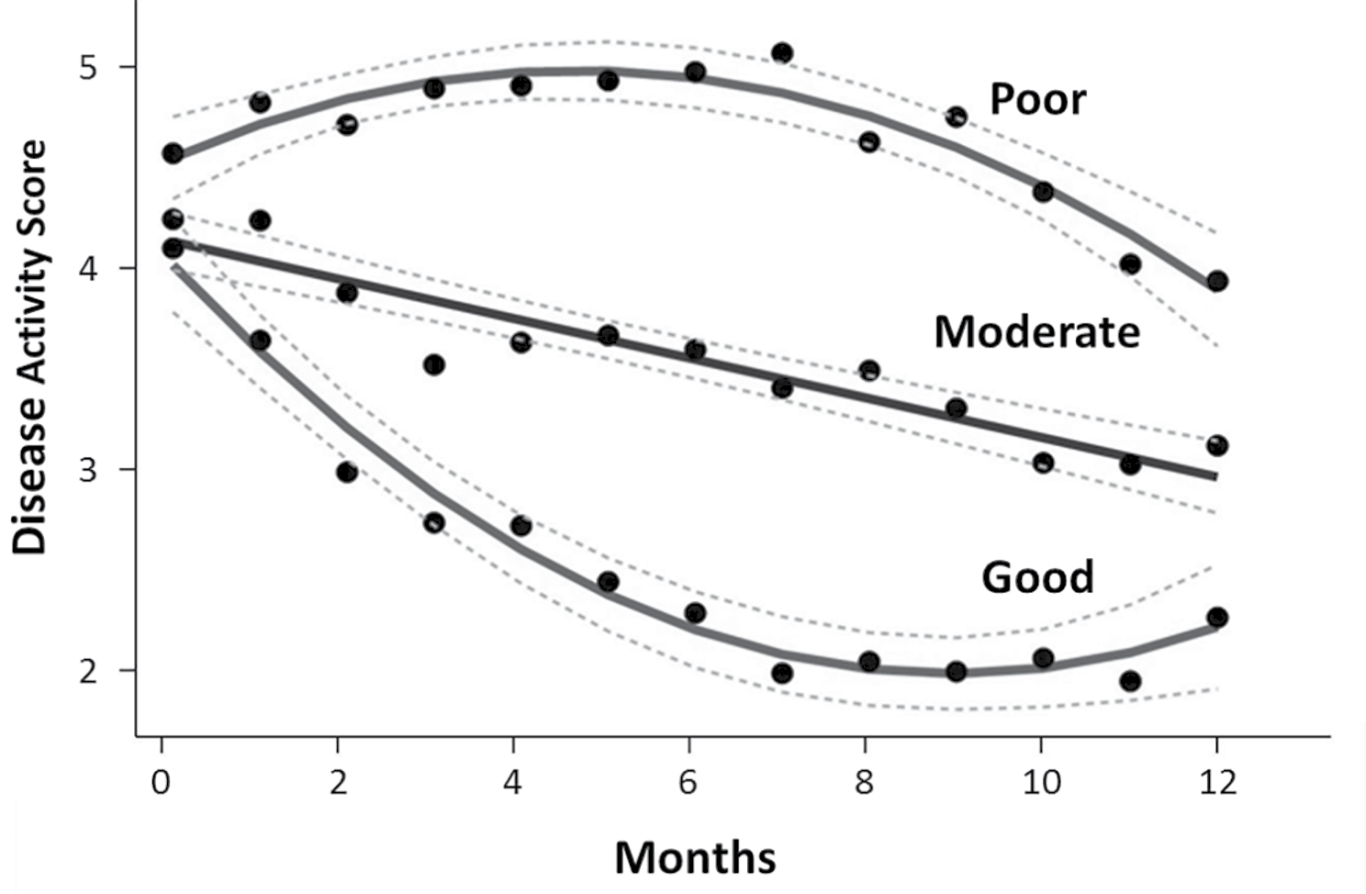
Mean Disease Activity Profiles Over 12 Months In The Three Group Based Trajectory Models (Good, Moderate And Poor Responders) Means and 95% confidence intervals are shown.

The 163 patients receiving intensive management were categorised into three groups based on changes in monthly DAS28-ESR scores over 12 months analysed using GBTMs (Fig. 1). These groups were termed good responders (n = 40), moderate responders (n = 76) and poor responders (n = 47). Figure 1, displays the shape of progression of DAS28-ESR scores over 12 months of follow up for the three groups.

**Fig. 2:**
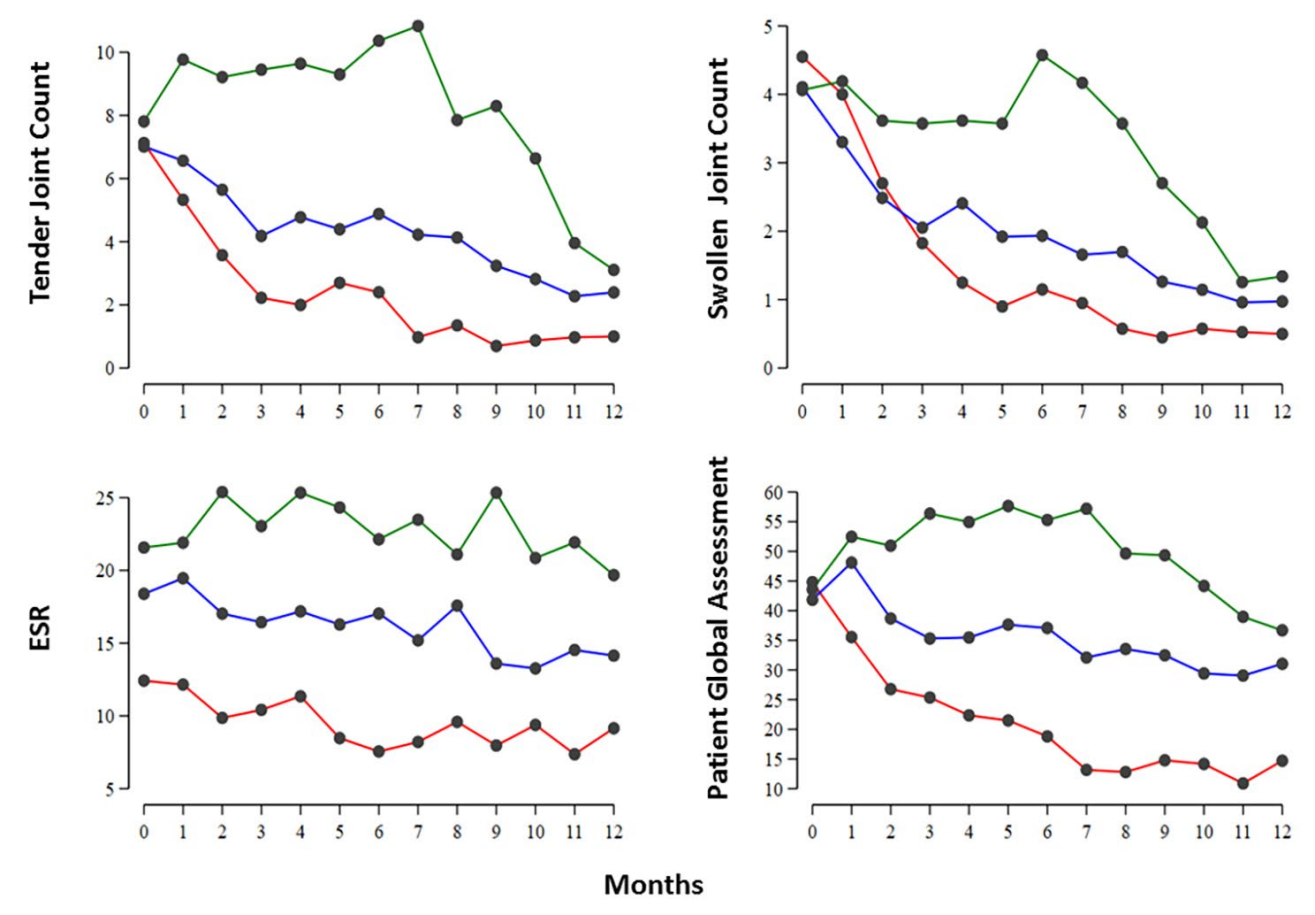
Mean 12-Month Profiles Stratified In The Three Group Based Trajectory Models (Good, Moderate And Poor Responders) Good responders – red (26%); moderate responders – blue (45%); poor responders – green (29%). Black circles are mean scores.

Changes in the individual components of DAS28-ESR scores showed a broadly similar pattern to changes in the composite score (Fig. 2). They all fell mostly in the good responders with the exception of the ESR, which was consistently lower in good responders but changed relatively little over time.

### Baseline data

Table 1 shows overall characteristics including ethnicity, age and disease duration were similar between the groups; there was a small, non-significant, excess of males in good responders.

**Table 1:** Baseline Data For The Three Responder Groups

		Total	Group			Significance
			*Good*	*Moderate*	*Poor*	
		*N = 163*	*N = 40*	*N = 76*	*N = 47*	
Sex, n (%)	Male	28 (17%)	10 (25%)	12 (16%)	6 (13%)	NS
	Female	135 (83%)	30 (75%)	64 (84%)	41 (87%)	
Ethnicity, n (%)	White	151 (93%)	38 (95%)	71 (93%)	42 (89%)	NS
	Black	6 (4%)	0 (0%)	3 (4%)	3 (6%)	
	Asian	3 (2%)	1 (3%)	2 (3%)	0 (0%)	
	Mixed	1 (1%)	1 (3%)	0 (0%)	0 (0%)	
	Other ethnic group	2 (1%)	0 (0%)	0 (0%)	2 (4%)	
Smoking, n (%)	Never smoked	56 (34%)	17 (43%)	24 (32%)	15 (32%)	NS
	Ever smoked	104 (64%)	22 (55%)	51 (67%)	31 (66%)	
	Missing	3 (2%)	1 (3%)	1 (1%)	1 (2%)	
Age (Years) Mean (SD)		56.2 (12.1)	55.3 (13.3)	57.3 (12.1)	55.3 (11.2)	NS
Disease Duration (Years) Mean (SD)		6.6 (6.8)	6.1 (5.6)	6.5 (6.1)	7.2 (8.6)	NS
BMI Mean (SD)		28.8 (8.6)	25.1 (4.7)	28.7 (6.2)	32.1 (12.5)	< 0.001
DAS28-ESR Mean (SD)		4.4 (0.5)	4.2 (0.6)	4.3 (0.5)	4.7 (0.4)	< 0.001
Tender Joint counts (28 joints) Mean (SD)		7.3 (4.0)	7.1 (3.9)	7.0 (4.4)	7.8 (3.5)	NS
Swollen joint counts (28 joints) Mean (SD)		4.2 (3.0)	4.6 (3.8)	4.1 (2.8)	4.1 (2.6)	NS
Erythrocyte Sedimentation Rate (mm/hr) Mean (SD)		17.9 (13.9)	12.4 (9.9)	18.4 (15.4)	21.6 (13.2)	0.008
C-Reactive Protein (mg/L) Mean (SD)		8.4 (10.6)	5.9 (6.8)	9.9 (13.2)	8.2 (8.0)	NS
Assessor Global Rating (mm) Mean (SD)		39 (18)	40 (22)	37 (18)	42 (15)	NS
Patient Global Assessment (mm) Mean (SD)		43.1 (19.1)	44.8 (22.6)	41.8 (18.9)	43.6 (16.3)	NS
HAQ Mean (SD)		1.2 (0.7)	0.9 (0.6)	1.3 (0.7)	1.5 (0.6)	< 0.001
Pain (mm) Mean (SD)		40 (23)	37 (26)	40 (23)	44 (19)	NS
Fatigue (mm) Mean (SD)		59 (25)	48 (30)	61 (23)	64 (21)	0.006
Patient Health Questionnaire-9 Mean (SD)		8.5 (6.1)	6.4 (4.9)	8.2 (5.9)	10.7 (7.0)	0.004

Significance tests are Chi-Squared for categorical data and One Way ANOVA for numerical data

Good responders had a significantly lower mean DAS28-ESR with relatively few scores above 4.5. The only individual component of the DAS28-ESR which was significantly different between groups was the ESR; it was lower in good responders. Other assessments of disease activity like C-reactive protein and assessors’ global rating, did not differ between groups.

There was a significant difference in BMI; it was highest in the poor responders. Obesity (BMI > 30) was present in 4/40 (10%) of good responders, 30/76 (38%) moderate responders and 20/47 (43%) of poor responders (*P* = 0.002 on Chi Square testing). Three other measures were significantly different between groups. HAQ scores and fatigue were lower in good responders together with PHQ-9 scores. Depression (PHQ-9 ≥ 15) occurred in 3/39 (8%) good responders, 11/76 (14%) moderate responders and 18/47 (38%) poor responders (*P* < 0.001 on Chi-Square testing).

### Drug treatments

There was no evidence that baseline drug treatments were different between groups; all patients were taking at least one initial conventional DMARD.

In total, 137/163 (84%) had one additional conventional DMARD, 63/163 (39%) had two additional conventional DMARDs and 4/163 (2%) had three additional conventional DMARDs (Table 2), and there were no significant differences among the groups. Similarly, there were no differences between groups in the numbers of patients increasing or decreasing doses of conventional DMARDs. In total, 69/163 (42%) increased their dose and 15/163 (9%) reduced their dose. Finally, there was also no difference in oral steroid use between groups; 15/163 (9%) received oral steroids. There was a trend for poor responders to receive more steroid injections.

**Table 2:** Treatments And Outcomes For The Three Responder Groups

	Total	Group			Significance
		*Good*	*Moderate*	*Poor*	
***Drug Treatments***					
One DMARD	137/163 (84%)	34/40 (85%)	64/76 (84%)	39/47 (83%)	NS
Two DMARDs	63/163 (39%)	10/40 (25%)	35/76 (46%)	18/47 (38%)	NS
Three DMARDs	4/163 (3%)	0	3/76 (4%)	1/47 (2%)	NS
Steroid Injections	70/163 (43%)	13/40 (33%)	31/76 (41%)	26/47 (55%)	NS
One Biologic	49/163 (30%)	2/40 (5%)	23/76 (30%)	24/47 (51%)	< 0.001
Two Biologics	7/163 (4%)	1/40 (3%)	2/76 (3%)	4/47 (9%)	NS
Three Biologics	2/163 (1%)	0	1/76 (1%)	1/47 (2%)	NS
***Management Approaches***					
Attended 8 or more monitoring visits	139/161 (86%)	34/40 (85%)	65/74 (88%)	40/47 (85%)	NS
Followed management algorithm at each visit	1122/1469 (76%)	330/373 (88%)	519/681 (76%)	273/415 (66%)	< 0.001
Pain management at each visit	1055/1533 (69%)	240/387 (62%)	501/708 (71%)	314/438 (72%)	0.004
Fatigue management at each visit	825/1533 (54%)	200/387 (52%)	384/710 (54%)	241/436 (55%)	NS
***End-Point Outcomes***					
DAS28-ESR Change, Mean (SD)	1.0 (1.4)	2.1 (0.8)	0.8 (1.3)	0.3 (1.2)	< 0.001
DAS28-ESR Remission, Number (%)	48/147 (29%)	28/36 (70%)	18/67 (24%)	2/44 (4%)	< 0.001
HAQ Change, Mean (SD)	0.2 (0.5)	0.4 (0.5)	0.2 (0.6)	0.1 (0.4)	NS
HAQ < 1.0	60/140 (42%)	25/33 (66%)	31/65 (41%)	12/42 (26%)	< 0.001
Pain Change, Mean (SD)	13 (32)	27 (27)	12 (31)	2 (32)	0.002
Pain < 20	80/148 (49%)	32/36 (80%)	37/68 (54%)	11/44 (24%)	< 0.001
Fatigue Change, Mean (SD)	18 (32)	27 (30)	21 (32)	6 (30)	0.008
Fatigue < 20	51/148 (31%)	23/36 (68%)	22/68 (29%)	6/44 (13%)	< 0.001

Significance tests are Chi-Squared for categorical data and One Way ANOVA for numerical data

There was a significant difference in biologic use. A first biologic was received by 2/40 (5%) of good responders, 23/76 (30%) moderate responders and 24/47 (51%) poor responders (*P* < 0.001). Only a few patients received second or third biologics with no significant differences between groups.

### Management approaches

Table 2 shows there were no differences between groups in patients’ attendance for monitoring visits; 139/163 (86%) patients attended eight or more visits. However, there were differences in adherence to the treatment algorithm: it was followed in 330/373 (88%) visits in good responders, 519/681 (76%) moderate responders and 273/415 (66%) poor responders (*P* < 0.001). There were no differences between groups in the reasons for these decisions. Overall, 48% of decisions not to follow the algorithm involved patient choice, 5% involved adverse events, 4% involved inter-current illness and 43% involved clinical discretion.

There was no difference between groups in fatigue management approaches; 52-55% of visits involved advice on fatigue. However, there were significant differences in advice on pain: 249/387 (62%) visits in good responders involved advice on pain management compared with 501/708 (71%) in moderate responders and 314/438 (72%) in poor responders (*P* = 0.004).

Three other measures were significantly different between groups. HAQ scores and fatigue were lower in good responders together with PHQ-9 scores. Depression (PHQ-9 ≥ 15) occurred in 3/39 (8%) good responders, 11/76 (14%) moderate responders and 18/47 (38%) poor responders (*P* < 0.001 on Chi-Square testing).

### End-point outcomes

Table 2 shows that mean DAS28-ESR changes and the numbers of patients in DAS28-ESR remission differed between groups with good responders achieving greater changes and more remissions than other groups. These differences reflect the construction of the groups.

There was a trend for mean HAQ changes to be greatest in the good responders, but this difference was not significant. However, significantly more good responders achieved HAQ scores < 1.0 than in other groups (66% vs. 41% and 26%).

Changes in pain and fatigue and the numbers of patients achieving end-point pain and fatigue scores < 20 were significantly different between groups. In each case the end-point outcomes were best in the good responders and worse in the poor responders.

### Multivariate modelling

The multinomial logistic model showed only some predictors of response groups were independent of each other. A model incorporating demographic variables (age, gender, ethnicity, smoking history and disease duration), baseline body mass index (BMI), baseline PHQ-9 and change in HAQ, pain, and fatigue showed only three factors acted independently (Table 3). In moderate responders, only changes in pain at 12-months were significant at the 5% level when compared to good responders. In poor responders there were significant associations with changes in pain at 12 months, and also baseline BMI and PHQ-9.

**Table 3:** Multinomial Logistic Model For Response Groups

	Response Groups				
	Good	Moderate		Poor	
		*Coefficient* *(95% CI)*	*p-value*	*Coefficient* *(95% CI)*	*p-value*
Pain Change	Reference	0.02 (0.01,0.04)	0.007	0.03 (0.01,0.05)	0.002
Patient Health Questionnaire (PHQ-9)	Reference	0.06 (-0.02,0.14)	0.130	0.13 (0.04,0.22)	0.005
Baseline BMI (kg/m^2^)	Reference	0.08 (-0.01,0.17)	0.066	0.14 (0.05,0.23)	0.004

CI = confidence intervals

## Discussion

Our analysis shows that it is practical and relevant to classify patients with RA receiving intensive management for moderately active RA using latent class modelling into different groups. When this approach is taken patients do not form two clearly defined groups of responders and non-responders; instead GBTMs identified three different developmental trajectories over the 12 months of the trial. These groups are likely to provide approximations for a more complex reality of clusters of patients following similar trajectories over time. It is probable that there are clinically important subpopulations of patients with RA that characterise longitudinal changes in disease activity. About one quarter of patients responded very well; another quarter did not respond at all; and about half showed moderate responses. Most good responders had endpoint remissions with low disability, pain and fatigue scores. Few poor responders achieved any favourable outcomes. There were important differences between groups both in their baseline predictors and in their relationship to different components of intensive management.

The good responders had the lowest baseline mean DAS28-ESR, ESR and BMI scores and only 10% were obese and 8% had depression. Only a few (5%) of the good responders required biologic treatments, suggesting patient-related factors are most important in determining response to treatment. Additional biologics were not needed in most good responders and the use of biologics was, consequently, not related to achieving remission. These findings reflect trial evidence about the value of intensive combination DMARDs provided by trials such as RACAT and TACIT [17, 18]. Although good responders followed the treatment algorithm most closely, this probably reflects the relative ease of doing so when patients responded to treatment. The decline in DAS28-ESR in these patients was apparent by three months. The TITRATE trial used DAS28-ESR to assess response because it was undertaken in English centres which traditionally use this assessment. It is possible that using C-reactive protein (CRP) in place of the ESR and constructing the DAS28-CRP may have given somewhat different findings. However, baseline and endpoint comparisons of DAS28-ESR and DAS28-CRP in the TITRATE trial did not show any clinically relevant differences between these measures [10].

The poor responders, who failed to show any reduction in DAS28-ESR and rarely achieved remission, reflect the concept of difficult to treat RA proposed by de Hair et al. [19]. However, as they had yet to fail two biologics, they could not meet the most recent EULAR criteria for this classification [20]. A recent systematic review by Roodenrijs et al. [21] concluded that the heterogeneity between individual patients with difficult to treat RA suggests a range of different pathogenic mechanisms are involved. Obesity was one factor, and it has been identified in a number of previous studies [22, 23]. High initial HAQ scores have also been associated with poor responses and more flares [24, 25]: Goetz et al. [24] reviewed 30 studies and found baseline HAQ scores were consistently associated with treatment responses; Bechman et al. [24] evaluated a single study and found the association between high HAQ scores and subsequent flares persisted when baseline DAS scores were taken into account in an adjusted analysis. Another factor which we found predicted poor responses was baseline depression; this has been identified previously in trials and observational studies [26–28]. Currently depression is rarely measured in either routine care or research settings.

Many trials compare treatments using a binary endpoint outcome, such as whether patients achieved remission. Using trajectories provides the opportunity to undertake a more nuanced assessment. Increasing both the numbers of good and moderate responders identified using GBTMs may be equally clinically relevant as simply increasing the numbers of patients achieving remission. Consequently, using GBTMs to assess response may identify differences in treatments which show superficially similar efficacy using binary outcome assessments.

Our study has a number of strengths. First, it involved patients from multiple English centres, and its findings are therefore likely to be generalisable. Secondly, it adopted a novel model-based approach which allowed formal identification of homogenous subgroups that is more flexible than the traditional binary division of response/no-response. Thirdly, the large number of repeated measures for each participant, and the use of an approach that met all the statistical criteria proposed for the assessment of good classification, shows the analysis was robust. Fourthly, the technical evidence provided by the statistics used such as BIC and Entropy, the posterior group membership probabilities were supportive of the classification achieved. Finally, the characteristics of the three groups indicate differences in baseline risks that are likely to have predicted the response to treatment at follow-up.

The study also has a number of limitations. Firstly, it had a relatively small sample size. However, there was substantial follow up data at 12 months and assessments of DAS28-ESR were made monthly with minimal missing data; the extensive repeated-measures data partially compensates for the relatively small number of participants [14]. Secondly, although the TITRATE trial had a control group, they were not assessed monthly and therefore GBTMs could not be used to assess their responses. If trajectories are to be used to test hypotheses in clinical trials both active and control groups will need to be followed with similar regular assessments, such as monthly DAS28-ESR scores. Finally, our results will need replicating in additional, potentially larger studies, to validate using GBTMs to assess trial outcomes in RA.

## Conclusions

We conclude that GBTMs identified three trajectories of disease progression in patients with moderate RA treated intensively. Several baseline variables influenced membership of different trajectories such as depression and obesity. These findings raise the possibility that patients with RA with co-morbid depression and obesity may need additional or different treatment approaches. Such a possibility merits further research in larger groups of patients. It would also be relevant to explore the relationship of other patient related outcomes to GBTMs.

## Data Availability

The data generated during this trial is not publicly available because consent to make their data publicly available was not specifically sought from trial participants. Anonymised summary data will be available from the corresponding author, Dr Fowzia Ibrahim (fowzia.ibrahim@kcl.ac.uk) for inclusion in meta-analyses and other relevant similar academic endeavours.

## List of Abbreviations

RA: Rheumatoid Arthritis
EULAR: European Alliance of Associations for Rheumatology
ACR: American College Of Rheumatology
DAS28-ESR: Disease Activity Score For 28 Joints With Erythrocyte Sedimentation Rate
DMARDs: Conventional Disease-Modifying Anti-Rheumatic Drugs
IM: Intra-Muscular
HAQ: Health Assessment Questionnaire
PHQ9: Patient Health Questionnaire-9
BMI: Body Mass Index
CRP: C-Reactive Protein
SD: Standard Deviations
GBTMs: Group Based Trajectory Models
